# 气相色谱-三重四极杆质谱法测定植物源性食品中棉隆及其代谢物异硫氰酸甲酯残留

**DOI:** 10.3724/SP.J.1123.2021.12021

**Published:** 2022-07-08

**Authors:** Jiefeng RONG, Meizhu XU, Zhiyong ZHANG, Qiang ZOU, Dunming XU, Jianhai ZHONG, Songyan ZHANG, Youdong LE, Ziwen SHE

**Affiliations:** 1.泉州海关综合技术服务中心, 福建 泉州 362000; 1. Quanzhou Customs Comprehensive Technology Service Center, Quanzhou 362000, China; 2.厦门海关技术中心, 福建 厦门 361000; 2. Xiamen Customs Technology Center, Xiamen 361000, China

**Keywords:** 气相色谱-三重四极杆质谱法, 棉隆, 异硫氰酸甲酯, 植物源性食品, gas chromatography-triple quadrupole mass spectrometry (GC-MS/MS), dazomet, methyl isothiocyanate, plant-derived foods

## Abstract

基于气相色谱-三重四极杆质谱建立了同时快速测定植物源性食品中棉隆及其代谢物异硫氰酸甲酯残留量的方法。对样品前处理及色谱条件进行了优化,蔬菜、水果、谷物、坚果、茶叶和香辛料样品经乙酸乙酯提取,离心后上清液经乙二胺-*N*-丙基硅烷化硅胶、石墨化炭黑、十八烷基键合硅胶和无水硫酸镁分散固相萃取净化,经DB-17MS色谱柱(30 m×0.25 mm×0.25 μm)分离,采用EI源,在多反应监测(MRM)模式下检测,基质匹配外标法定量。在优化实验条件下,目标化合物在0.005~1 mg/L范围内的线性关系良好,相关系数均大于0. 99,方法定量限为0.01 mg/kg。空白样品在4个添加水平下的平均回收率为74.2%~117.2%,相对标准偏差(*n*=6)为2.8%~9.0%,方法的准确度和精密度符合农药残留测定要求。应用建立的方法对实验室日常样品大白菜、韭菜、豇豆、油麦菜、茄子、姜、芹菜、马铃薯、橙、猕猴桃、西红柿、辣椒、大米、茶叶、杏仁和孜然各6个进行检测,均未检出目标物。该方法具有操作简单、快速、灵敏、准确的特点,解决了已有方法中需采用两种前处理方法、两套设备进行检测的不足,能满足植物源食品中棉隆及其代谢物异硫氰酸甲酯的检测要求。

## Abstract (en)

Dazomet is a kind of crystal solid that is stable at room temperature and acts as a fumigant. It is commonly used to control soil fungi, as an insecticide, and in sterilization and weeding. It can effectively kill root-knot nematodes, soil pests, weeds, and many soil-borne disease-causing organisms, to provide clean and healthy soil. Dazomet slowly decomposes and releases methyl isothiocyanate, methylamine, carbon disulfide, and hydrogen sulfide in acidic soil, and diffuses upward through the spaces in the soil to kill contact organisms. When agricultural crops are planted in soil treated with cotton wool, the residues in the grown crop can cause harm to human body when consumed. To ensure the quality and safety of food crops, it is important to develop a detection method for dazomet and its metabolites in plant-derived foods. Hence, in this study, a rapid and simultaneous determination method was developed for dazomet and its metabolite methyl isothiocyanate residues in plant-derived foods by gas chromatography-triple quadrupole mass spectrometry (GC-MS/MS). The sample pretreatment and chromatographic conditions were optimized in the experiment. Subsequently, dazomet and its metabolite methyl isothiocyanate residues in vegetables, fruits, grains, nuts, tea, and spices were extracted with ethyl acetate, and purified using graphitized carbon, a primary-secondary amine, stearyl-bonded silica gel, and anhydrous magnesium sulfate as dispersive solid-phase extraction sorbents. After centrifugation and filtration, the target compounds were analyzed in the multiple reaction monitoring (MRM) mode by GC-MS/MS, and quantified by matrix matching external standard method. The matrix effects of the samples were also evaluated. The matrix effect was found to be in the range of 2.5% to 13.6% for methyl isothiocyanate in 16 matrices. As this matrix effect was weak, there was no need for compensatory measures. In contrast, the matrix effect of dazomet in 16 matrices was in the range of 240.3% to 331.2%. This matrix effect was strong and required compensation. Finally, a matrix matching calibration method was used to compensate the matrix effects. The relative matrix effects of other tested substrates were analyzed using lettuce as the representative substrate; it was found that all showed weak matrix effects. Therefore, the use of lettuce as a representative matrix to prepare a matrix standard curve can effectively correct the matrix effects of dazomet and methyl isothiocyanate in other substrates. Under the optimal conditions, the calibration curves were linear in the range of 0.005-1 mg/L with correlation coefficients higher than 0.99. Recovery tests were conducted by adding mixed standards to blank samples at four levels. The recoveries were in the range of 74.2%-117.2% with relative standard deviations (RSDs, *n*=6) of 2.8%-9.0%. The limits of quantification (LOQs) of dazomet and methyl isothiocyanate were 0.01 mg/kg. The accuracy and precision of this method met the requirements of pesticide residue determination. The established method was used to detect dazomet and its metabolite methyl isothiocyanate residues in six samples of Chinese cabbage, Chinese chives, cowpea, lettuce, eggplant, ginger, celery, potato, orange, kiwifruit, tomato, chili, rice, tea, almond, and *Cuminum cyminum* L. in the laboratory, and nothing was detected. The method is simple, rapid, and sensitive; overcomes the shortcomings of existing methods that require two pretreatment steps and two sets of equipment; and meets the requirements for the detection of dazomet and its metabolite methyl isothiocyanate residues in plant-derived foods.

棉隆,化学名称为3,5-二甲基-1,3,5-噻二嗪-2-硫酮,是一种室温下稳定的晶体固体,具有熏蒸作用,常用于控制土壤真菌、杀虫、杀菌和除草,能有效杀灭根结线虫、土壤害虫、杂草及多种土传病害,从而获得洁净及健康的土壤;棉隆是替代溴甲烷较理想的一种土壤处理剂^[1,2]^。棉隆在酸性土壤中会缓慢分解释放异硫氰酸甲酯、甲胺、二硫化碳和硫化氢,通过土壤中的空间向上扩散,杀死与之接触的生物^[3]^。在经棉隆处理后的土壤中种植农产品时,会引发食品中残留从而造成对人体的危害,各国政府均制定了棉隆的残留限量进行监控。日本和欧盟对棉隆的残留定义都是以异硫氰酸甲酯来计,日本对薯类、蔬菜、水果、香草类等植物源性食品中制定了50余个残留限量值,残留限量范围为0.01~2 mg/kg,欧盟对谷物、坚果、茶叶、香辛料、蔬菜、水果等植物源性食品中制定了280余个残留限量,残留限量范围为0.02~0.15 mg/kg。目前,棉隆在我国的番茄、草莓、姜和菊科4种作物上有登记,我国食品安全国家标准GB 2763-2021《食品安全国家标准食品中农药最大残留限量》中棉隆的残留定义为棉隆及其代谢物异硫氰酸甲酯之和,并制定了番茄和姜中的临时限量为0.02 mg/kg和2 mg/kg^[4]^。因此,为确保食品质量安全,开发植物源性食品中棉隆及异硫氰酸甲酯测定方法具有极其重要的作用。

文献报道的棉隆及异硫氰酸甲酯测定方法主要有液相色谱(LC)测定棉隆^[5]^;使用气相色谱(GC)^[6]^、气相色谱-质谱(GC-MS)^[6]^、LC^[7]^和气相色谱-三重四极杆质谱(GC-MS/MS)^[8]^测定异硫氰酸甲酯;相关方法仅测定棉隆和异硫氰酸甲酯中的一种。此外,冯义志等^[9,10]^建立了采用LC测定番茄和土壤中棉隆、GC-MS测定番茄和土壤中异硫氰酸甲酯的方法;徐峰等^[11]^建立了采用超高效液相色谱-三重四极杆质谱法测定草莓中棉隆、GC测定草莓中异硫氰酸甲酯残留的检测方法。相关文献报道中均未实现使用一种前处理方法和同一种仪器设备对棉隆及异硫氰酸甲酯同时测定。因此,本研究拟使用一种前处理方法和同一种仪器设备,建立简单、快速、准确的植物源性食品中棉隆及其代谢物异硫氰酸甲酯残留量的同时检测方法。近年来,相较于常规的单四极杆GC-MS仪,气相色谱-三重四极杆质谱联用仪因其抗干扰能力强和灵敏度高等优点而广泛应用于蔬菜、水果、粮谷、茶叶、药材中农药、兽药、非法添加物等的检测中^[12⇓⇓⇓-16]^。

本研究植物源性食品中残留的棉隆及其代谢物异硫氰酸甲酯经乙酸乙酯提取,提取液经分散固相萃取净化,建立了棉隆及其代谢物异硫氰酸甲酯的气相色谱-三重四极杆质谱检测方法。建立的方法操作简单便捷,能满足植物源性食品中残留的棉隆及其代谢物异硫氰酸甲酯的检测要求。

## 1 实验部分

### 1.1 仪器、试剂与材料

Agilent 7890A-7000B气相色谱-三重四极杆质谱联用仪(美国安捷伦公司):配备电子轰击(EI)源。无水硫酸镁(MgSO_4_)、氯化钠、丙酮、正己烷和乙醚均为分析纯,购于国药集团化学试剂有限公司;乙酸乙酯(色谱纯)购于美国TEDIA公司。乙二胺-*N*-丙基硅烷化硅胶(PSA, 40~60 μm)、石墨化炭黑(GCB, 40~120 μm)和十八烷基键合硅胶(C_18_, 40~60 μm)购于上海安谱实验科技股份有限公司。棉隆(C_5_H_10_N_2_S_2_,纯度99.8%)和异硫氰酸甲酯(C_2_H_3_NS,纯度98.0%)购于广州佳途科技股份有限公司。

### 1.2 标准溶液配制

准确称取适量棉隆和异硫氰酸甲酯标准品,分别用乙酸乙酯配制成质量浓度为1000 mg/L的标准储备液;分别吸取适量的上述标准储备液,用乙酸乙酯配制成10 mg/L的混合标准中间液。

吸取适量的混合标准中间液,用空白样品提取液(空白样品按1.3节处理)稀释成质量浓度为5、10、50、200和1000 μg/L的基质混合标准工作溶液,基质混合标准工作溶液应现配现用。

所有标准溶液均避光于-18 ℃以下保存。

### 1.3 样品前处理

#### 1.3.1 蔬菜和水果

称取10 g(精确至0.01 g)试样于50 mL塑料离心管中,加入4 g氯化钠、10 mL乙酸乙酯,涡旋振荡提取5 min; 4000 r/min离心3 min。准确吸取1.5 mL上清液于2 mL聚丙烯离心管中,加入50 mg GCB、50 mg PSA、50 mg C_18_和50 mg MgSO_4_,涡旋混合1 min, 14000 r/min离心3 min,取上清液过0.22 μm有机滤膜,待测。

#### 1.3.2 茶叶、谷物、坚果和香辛料

称取5 g(精确至0.01 g)试样于50 mL塑料离心管中,加入10 mL乙酸乙酯,涡旋振荡提取5 min; 4000 r/min离心3 min。准确吸取1.5 mL上清液于2 mL聚丙烯离心管中,谷物和坚果提取液中加入50 mg GCB、50 mg PSA、50 mg C_18_和50 mg MgSO_4_,茶叶和香辛料提取液中加入125 mg GCB、50 mg PSA、50 mg C_18_和50 mg MgSO_4_,涡旋混合1 min, 14000 r/min离心3 min,取上清液过0.22 μm有机滤膜,待测。

### 1.4 仪器条件

DB-17MS毛细管色谱柱(30 m×0.25 mm×0.25 μm)柱;柱箱升温程序:35 ℃保持4 min,然后以10 ℃/min升温至50 ℃,保持2 min;再以50 ℃/min升温至230 ℃,保持2 min;最后以25 ℃/min升温至280 ℃,保持0 min;载气:氦气,纯度≥99.999%,恒流模式,流量为1.0 mL/min;进样口温度:280 ℃;进样量:1 μL;进样方式:不分流进样。

电子轰击源:70 eV;离子源温度:230 ℃;传输线温度:280 ℃;溶剂延迟:4.2 min;多反应监测(MRM)模式:棉隆和异硫氰酸甲酯分别选择一个定量离子对、一个定性离子对,其保留时间、定量离子对、定性离子对和碰撞能量见表1。

**表 1:** 棉隆和异硫氰酸甲酯的保留时间、定量离子对、定性离子对和碰撞能量

Analyte	Retention time/min	Quantitative analysis			Qualitative analysis	
		Ion pair	CE/eV		Ion pair	CE/eV
Dazomet	13.91	162.1>89.0	8		89.0>44.1	10
Methyl isothio- cyanate	4.91	73.1>71.9	20		71.9>45.0	10

## 2 结果与讨论

### 2.1 仪器条件的优化

#### 2.1.1 仪器的选择

在已有研究基础上,尝试了使用超高效液相色谱-三重四极杆质谱仪、气相色谱-质谱联用仪和气相色谱-三重四极杆质谱联用仪对植物源性食品中棉隆及其代谢物异硫氰酸甲酯进行同时检测。结果表明使用超高效液相色谱-串联质谱仪进行检测时棉隆的响应较好,但异硫氰酸甲酯无响应;使用气相色谱-质谱联用仪进行检测时,棉隆和异硫氰酸甲酯的响应均较好,但进行实际样品检测时容易受样品基质干扰,色谱峰容易出现峰展宽、峰拖尾和干扰峰等现象,导致定性困难,方法准确性无法满足要求;只有在气相色谱-三重四极杆质谱联用仪上棉隆和异硫氰酸甲酯的响应均良好,实际样品检测准确度、精密度等均可满足要求,最终确定使用气相色谱-三重四极杆质谱联用仪对植物源性食品中棉隆及其代谢物异硫氰酸甲酯进行同时检测。

#### 2.1.2 色谱柱的选择

从文献^[9⇓-11]^可知,可使用非极性、低流失的色谱柱DB-1(30 m×0.25 mm×0.25 μm)或者中等极性、低流失色谱柱TG-1701MS(30 m×0.25 mm×0.25 μm)分离异硫氰酸甲酯。实验比较了DB-5MS、DB-17MS、DB-1701 3种色谱柱对棉隆及异硫氰酸甲酯的分离效果,其中DB-5MS色谱柱为非极性色谱柱,极性与DB-1色谱柱相当;DB-17MS色谱柱为中等极性色谱柱;DB-1701色谱柱为低/中等极性色谱柱,其固定相及其极性和TG-1701MS色谱柱相当。实验结果表明棉隆和异硫氰酸甲酯在DB-5MS、DB-17MS、DB-1701 3种色谱柱上均可良好分离,主要区别在于DB-5MS色谱柱对异硫氰酸甲酯的保留能力较差,导致异硫氰酸甲酯在DB-5MS柱上出峰时间相对DB-17MS、DB-1701更早,从而可能受到溶剂峰的影响;最终,选择DB-17MS柱作为本方法的色谱柱。

#### 2.1.3 质谱条件的优化

选择10.0 mg/L的棉隆和异硫氰酸甲酯乙酸乙酯溶液进行MS全扫描,选择响应较高的离子作为母离子,确定棉隆的母离子为89.0和162.1,异硫氰酸甲酯的母离子为73.1和71.9。再对相应母离子进行产物离子扫描,其中棉隆响应较好的MRM离子对为162.1>89.0, 89.0>44.1, 162.1>44.1;异硫氰酸甲酯响应较好的MRM离子对为73.1>71.9, 71.9>45.0, 73.1>45.0;由于162.1>44.1和73.1>45.0的响应相对较差且容易受到基质的影响,所以放弃选择这两个离子对作为定性离子对。最终确定棉隆和异硫氰酸甲酯的定量及定性离子对见表1。0.1 mg/L基质混合标准溶液中棉隆和异硫氰酸甲酯的提取离子色谱图见图1。

**图 1:**
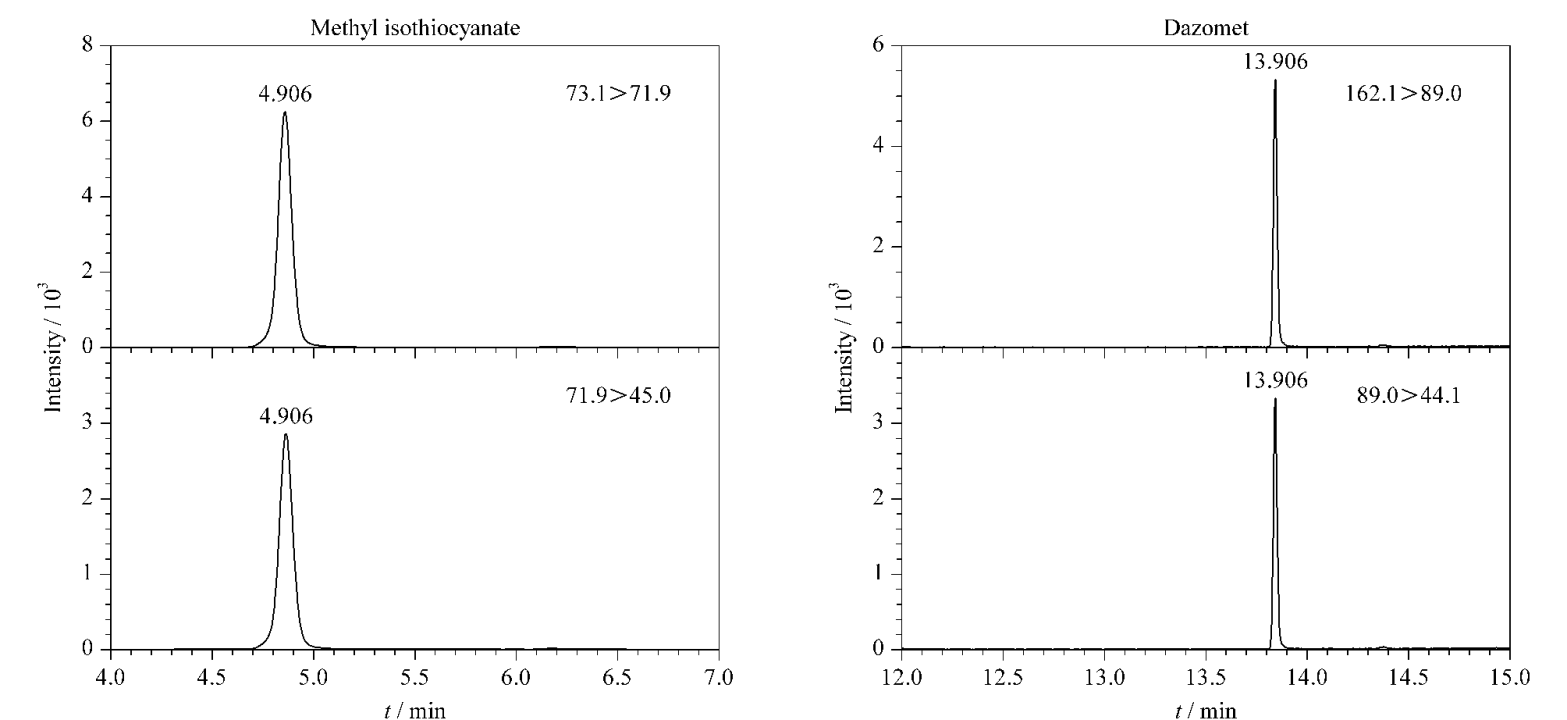
棉隆和异硫氰酸甲酯的提取离子色谱图

### 2.2 前处理方法的优化

#### 2.2.1 提取溶剂的选择

实验选择正己烷、丙酮、乙酸乙酯、乙醚配制1.0 mg/L棉隆和异硫氰酸甲酯混合标准溶液上机分析,结果表明棉隆在以上溶剂中的响应和峰形均较好,而异硫氰酸甲酯在乙酸乙酯和丙酮为溶剂时峰形较好,在正己烷和乙醚为溶剂时峰展宽、峰形差,所以前处理提取溶剂选择丙酮或者乙酸乙酯比较合适。因丙酮的极性较强,容易把更多的叶绿素等干扰物质提取出来,且丙酮相比乙酸乙酯在水中的溶解度更高,为降低后续净化处理的难度,最终选择使用乙酸乙酯为提取溶剂。

#### 2.2.2 净化试剂的选择

实验考察了GCB、C_18_、PSA、MgSO_4_ 4种物质对棉隆和异硫氰酸甲酯标准溶液进行吸附后的回收率。分别称取100 mg GCB、C_18_、PSA、MgSO_4_,对1 mL的棉隆和异硫氰酸甲酯标准溶液(1.0 mg/L)吸附情况进行考察。试验结果表明,PSA、C_18_、GCB、MgSO_4_对棉隆和异硫氰酸甲酯的吸附回收率都在90%~110%之间;说明4种物质均不会对棉隆和异硫氰酸甲酯造成吸附,因此,本方法使用GCB、PSA、C_18_除去提取液中的杂质、MgSO_4_除去提取液中的水分。其中GCB对样品中极性和非极性的有机化合物干扰物有极高的吸附力,对去除植物中色素有十分显著的效果;PSA对植物源样品基质中的有机酸、色素、糖类等都有较好的净化作用;C_18_可去除如挥发油、萜类、脂类等非极性化合物。

#### 2.2.3 吸附剂用量的优化

植物源样品种类繁多,基质也比较复杂,所以使用分散固相萃取净化时需要考虑两种或多种吸附净化剂,以较好地除去样品中的叶绿素、脂类和糖类等干扰仪器分析的杂质。实验复配了含量不同的3组吸附剂组合对代表性基质蔬菜(韭菜)、水果(橙)、谷物(大米)、油料(杏仁)、茶叶和香辛料(孜然)空白样品的0.20 mg/kg加标样提取液进行净化;提取净化实验步骤按照1.3节进行,考察不同组合吸附剂的净化效果和添加回收率。如图2所示,3种吸附剂组合下,异硫氰酸甲酯在6种基质中的回收率均在80%~110%之间,均可满足分析方法要求。棉隆在6种基质中的回收率均非常高,表现为强基质效应,随着净化试剂用量加大,回收率逐步下降但未降至满足分析方法要求的水平;为了提高分析方法的准确性,仍然需要采取其他方式补偿棉隆的基质效应。综合考虑净化效果、成本、便捷性、仪器分析时基线干扰,选择50 mg PSA+50 mg GCB+50 mg C_18_+50 mg MgSO_4_组合对蔬菜、水果、谷物和坚果进行净化,对于色素含量较多的茶叶和香辛料,适当加大GCB的用量,选择50 mg PSA+125 mg GCB+50 mg C_18_+50 mg MgSO_4_组合对茶叶和香辛料进行净化。

**图 2:**
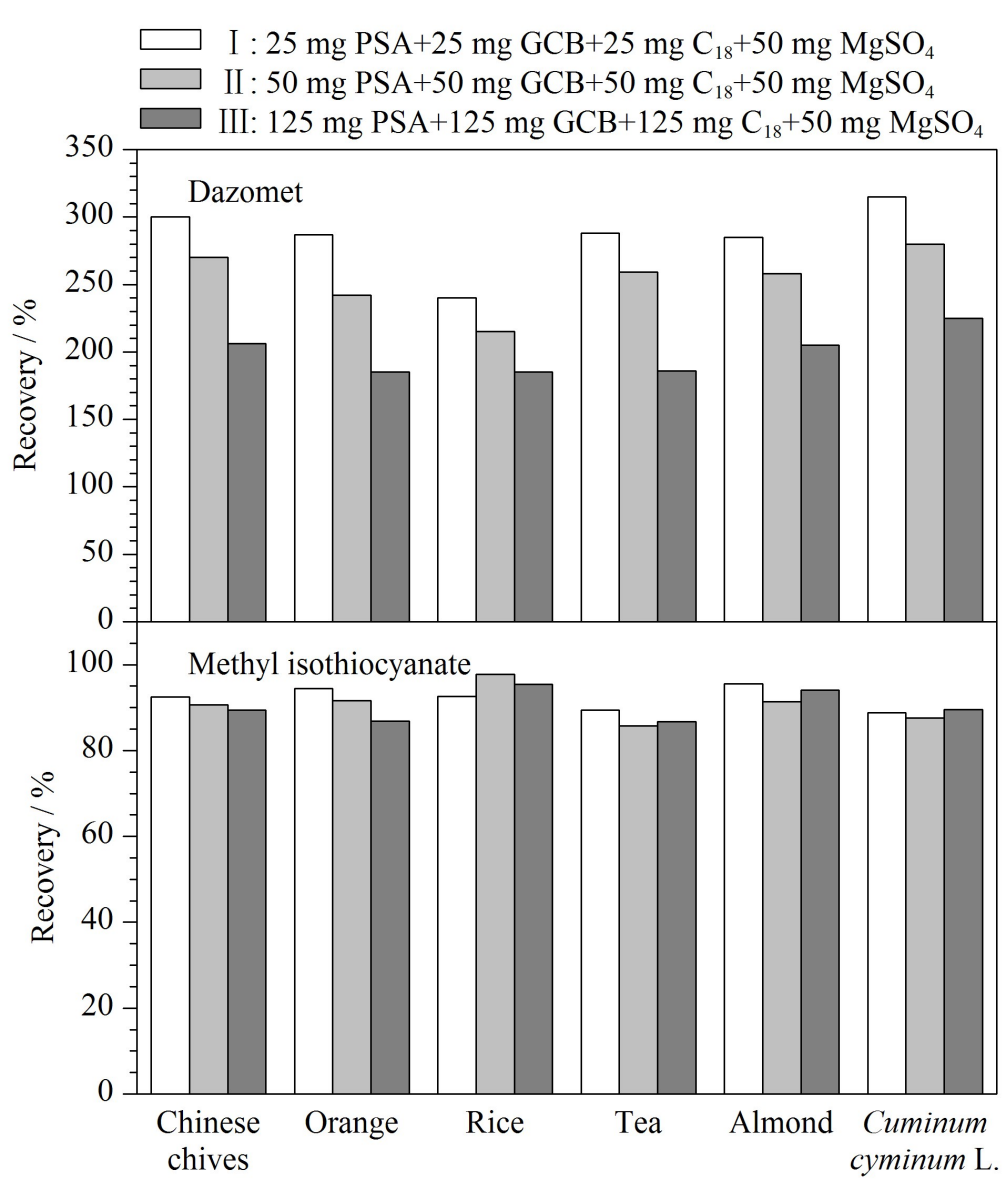
不同吸附剂组合对加标空白样品提取回收率的影响

### 2.3 基质效应(ME)的探究

在吸附剂用量的优化中可知,棉隆表现出强基质效应,且通过分散固相萃取净化的方式无法将其回收率降至合理范围,因此有必要对基质效应进行探究,以确保分析方法的准确性。试验按照1.3节的前处理方法制备空白基质溶液,按照1.2节的方法配制标准工作溶液和基质匹配标准工作溶液上机检测,按以下公式对基质效应进行评估:ME=[(基质匹配标准曲线斜率/纯溶剂标准曲线斜率)-1]×100%。|ME|<20%为弱基质效应,可忽略而无需采取补偿措施;20%≤|ME|≤50%为中等程度基质效应,|ME|>50%为强基质效应,须采取措施补偿基质效应^[17,18]^。异硫氰酸甲酯和棉隆的基质效应如图3所示,其中异硫氰酸甲酯在16种基质中基质效应均较小(ME值为2.5%~13.6%),无需采用措施补偿基质效应;但棉隆表现为强基质效应(ME值240.3%~331.2%),需采取措施补偿,本研究采用基质匹配校准法进行基质效应的补偿。

**图 3:**
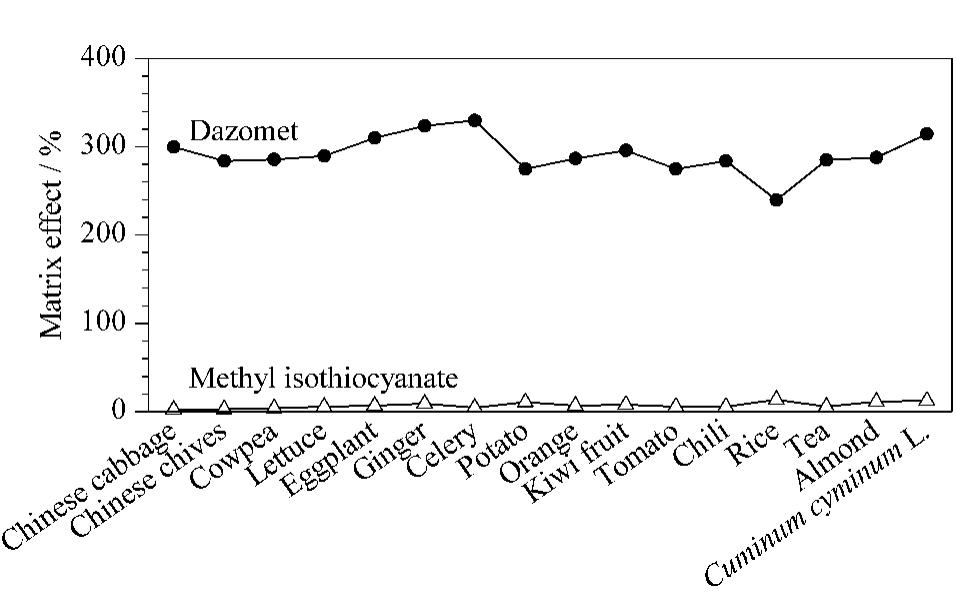
不同样品中棉隆和异硫氰酸甲酯的基质效应

我国关于植物源性食品农药残留检测的国家标准GB 23200.113-2018^[19]^及GB 23200.121-2021^[20]^等都采用了基质匹配标准溶液的定量方法进行定量分析,然而针对多种多样的样品基质,在配制基质匹配标准溶液的过程中,大大降低了检测效率。为提高检测效率,本研究对代表性基质的选择进行探讨^[21]^。从图3可知,油麦菜中棉隆的基质效应在16种基质中接近于中位值,按照以下公式计算不同基质相对油麦菜的基质效应:相对基质效应=[(基质匹配标准曲线斜率/油麦菜基质匹配标准曲线斜率)-1]×100%,不同样品中棉隆相对于油麦菜的基质效应如图4所示,结果表明棉隆在15种基质中相对油麦菜的基质效应均小于20%,表现为弱基质效应。因此,可以选择油麦菜为代表性基质配制基质匹配标准工作溶液以补偿棉隆的基质效应,从而有效校正待测农药在其他基质中的基质效应,通过此方法在保证定量准确性和稳定性的前提下,提高检测效率。

**图 4:**
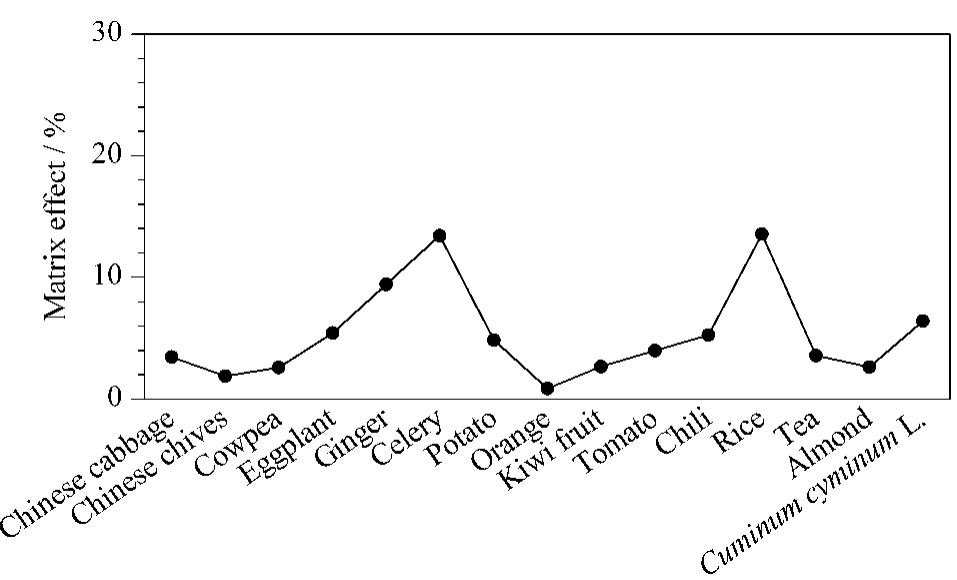
不同样品中棉隆的相对基质效应

### 2.4 线性范围、相关系数和定量限

用油麦菜空白基质溶液配制质量浓度为5、10、50、200和1000 μg/L的系列棉隆和异硫氰酸甲酯基质混合标准工作溶液,按1.4节仪器条件进行检测,以棉隆和异硫氰酸甲酯的质量浓度(*X*, μg/L)为横坐标,以棉隆和异硫氰酸甲酯的峰面积(*Y*)为纵坐标绘制基质标准工作曲线,得到线性方程和相关系数(*r*);在空白样品溶液中添加适量的标准溶液后上机测定,以*S/N*=10确定定量限(LOQ),相关数据见表2。

**表 2:** 棉隆和异硫氰酸甲酯的回归方程、相关系数、线性范围和定量限

Analyte	Regression equation	r	Linear range/(mg/L)	LOQ/(mg/kg)	
Dazomet	Y=9.450X-93.199	0.9993	0.005-1	0.01	
Methyl isothiocyanate	Y=301.487X-3783.693	0.9988	0.005-1	0.01	

Y: peak area of quantitative ion; X: mass concentration, μg/L.

### 2.5 回收率和精密度

分别对大白菜、韭菜、豇豆、油麦菜、茄子、姜、芹菜、马铃薯、橙、猕猴桃、西红柿、辣椒、大米、茶叶、杏仁和孜然空白样品进行了不同浓度的标准添加回收试验,每个加标水平做6个平行。棉隆及其代谢物异硫氰酸甲酯在这些植物源样品中不同添加水平的平均回收率和精密度见表3。

**表 3:** 棉隆和异硫氰酸甲酯在不同样品中4个水平下的平均加标回收率和相对标准偏差(n=6)

Analyte	Spiked/ (mg/kg)	Chinese cabbage				Chinese chives						Cowpea					Lettuce								Eggplant									Ginger					
		Rec./%	RSD/%			Rec./%			RSD/%			Rec./%			RSD/%		Rec./%				RSD/%				Rec./%			RSD/%						Rec./%					RSD/%
Dazomet	0.01	94.2	4.9			100.0			5.6			101.0			4.3		99.2				5.9				91.2			6.7						94.0					6.4
	0.02	100.6	6.4			89.4			6.5			100.4			2.9		92.1				6.9				90.0			6.2						108.9					7.4
	0.1	92.7	5.6			89.9			4.4			99.5			6.5		102.1				5.5				99.4			4.8						91.2					4.9
	1	93.8	3.4			92.5			4.1			98.7			3.6		95.6				4.5				92.5			4.3						91.6					4.2
Methyl isothio-	0.01	100.7	3.6			103.3			5.0			104.4			4.6		100.0				6.3				97.2			6.7						95.9					5.9
cyanate	0.02	94.6	4.5			91.8			6.5			88.0			6.5		85.0				4.8				90.5			5.9						87.6					7.1
	0.1	81.4	6.0			82.9			5.3			85.3			7.4		78.3				4.5				90.7			4.8						89.0					5.4
	1	92.5	5.2			83.4			5.4			88.4			5.2		84.6				5.7				89.5			3.9						92.5					5.1
Analyte	Spiked/ (mg/kg)	Celery						Potato						Orange									Kiwi fruit									Tomato							
		Rec./%		RSD/%				Rec./%		RSD/%				Rec./%					RSD/%				Rec./%				RSD/%					Rec./%							RSD/%
Dazomet	0.01	89.2		3.7				94.5		4.4				104.3					4.2				101.2				2.8					103.9							4.3
	0.02	104.3		5.0				104.9		5.5				115.2					6.9				110.7				8.2					100.8							6.1
	0.1	103.0		4.5				99.4		4.3				105.5					3.9				106.7				5.0					104.7							5.9
	1	95.6		4.6				95.8		4.0				101.5					4.8				105.6				4.8					98.6							4.9
Methyl isothio-	0.01	102.6		6.8				104.8		4.8				102.2					4.2				100.9				2.9					101.3							3.0
cyanate	0.02	89.6		6.9				82.4		8.1				90.3					3.9				89.2				5.8					92.0							4.0
	0.1	88.3		5.5				79.2		3.4				85.0					6.4				79.9				6.4					80.5							5.4
	1	93.4		5.4				87.5		4.7				86.9					5.7				85.4				4.2					91.5							5.3
Analyte	Spiked/ (mg/kg)	Chili						Rice						Tea										Almond								Cuminum cyminum L.							
		Rec./%		RSD/%				Rec./%		RSD/%				Rec./%			RSD/%							Rec./%				RSD/%				Rec./%							RSD/%
Dazomet	0.01	102.0		5.4				103.5		5.9				96.0					4.8				81.5				9					87.1							7.6
	0.02	117.2		4.3				89.4		5.9				84.7					4.4				84.0				5.4					87.0							3.3
	0.1	106.1		4.0				83.2		4.7				84.6					6.3				90.6				5.7					91.0							3.5
	1	104.5		4.7				95.2		4.9				92.5					4.7				85.6				5.6					87.5							5.2
Methyl isothio-	0.01	100.4		6.1				97.4		7.0				89.2					7.2				87.6				5.9					90.0							3.3
cyanate	0.02	89.6		6.4				92.4		5.6				84.5					4.8				90.0				4.1					85.0							4.5
	0.1	84.8		7.2				82.1		8.5				74.2					8.3				92.4				4.8					90.1							3.9
	1	86.5		5.6				85.8		6.5				81.5					5.2				91.8				5.1					88.9							4.5

Rec.: recovery.

### 2.6 实际样品检测

用本方法对实验室日常样品大白菜、韭菜、豇豆、油麦菜、茄子、姜、芹菜、马铃薯、橙、猕猴桃、西红柿、辣椒、大米、茶叶、杏仁和孜然各6个进行检测,检测结果均为未检出。

## 3 结论

本文建立了气相色谱-三重四极杆质谱法同时测定植物源性食品中棉隆及其代谢物异硫氰酸甲酯残留量的检测方法。与已有方法相比,本方法使用同一种前处理方法、同一台仪器完成对植物源性食品中残留的棉隆及其代谢物异硫氰酸甲酯的检测,该方法操作简单、灵敏度高、准确度好,能满足植物源性食品中残留的棉隆及其代谢物异硫氰酸甲酯的检测需求,可为植物源性食品中残留的棉隆及其代谢物异硫氰酸甲酯风险监控提供有效的技术支持。
